# A virtual patient model for students’ interprofessional learning in primary healthcare

**DOI:** 10.1371/journal.pone.0238797

**Published:** 2020-09-23

**Authors:** Carrie Tran, Eva Toth-Pal, Solvig Ekblad, Uno Fors, Helena Salminen

**Affiliations:** 1 Department of Neurobiology, Care Sciences and Society, Division of Family Medicine and Primary Care, Karolinska Institutet, Stockholm, Sweden; 2 Academic Primary Healthcare Centre, Region Stockholm, Stockholm, Sweden; 3 Department of Learning, Informatics, Management and Ethics, Cultural Medicine, Karolinska Institutet, Stockholm, Sweden; 4 Department of Computer and System Sciences, Stockholm University, Stockholm, Sweden; Leiden University Medical Center, NETHERLANDS

## Abstract

**Objectives:**

Interprofessional education is important for increasing the quality of patient care, but organising it in primary healthcare is still challenging. The aim of this study was to develop and assess a virtual patient model for primary healthcare and to investigate students' perceptions of learning with this interprofessional virtual patient model.

**Methods:**

The virtual patient case described a patient with several medical conditions who had returned home after surgery. The virtual patient included text files, short videos, and links to illustrate different health professions' roles in home care. Ten interprofessional groups with 39 students assessed the virtual patient from four different study programmes: nursing, physiotherapy, medicine, and occupational therapy. The students answered a questionnaire about how they perceived the usability of the virtual patient and participated in group interviews. Qualitative content analysis was used to analyse the data from the semi-structured group interviews.

**Results:**

The analysis of the interviews resulted in four main categories: *The virtual patient model facilitated the learning process; It was beneficial to have students from different programmes in the group when working with the virtual patient; Working with the virtual patient helped the students to understand the roles and competencies of their own and other professions* and *All professions are needed in clinical work in order to help the patient*. The students perceived that the mixture of text and multimedia made the virtual patient seem authentic and stimulated their group discussions, which they valued most. The students gave generally high points for usability in the questionnaire, but they also gave input for improvement of the program in their comments.

**Conclusions:**

The interprofessional virtual patient model facilitated interactions and discussions between students and may be a useful complement for interprofessional education in clinical contexts and might be a suitable tool in preparing students for future teamwork.

## Introduction

There is currently a need for greater interprofessional collaboration in all healthcare sectors, but this is especially the case in primary healthcare due to the multiple and complex demands of ageing populations with chronic diseases [1, 2]. All healthcare students need to learn to collaborate with other professions in order to be prepared to meet patients with all kind of needs. There exists an increasing body of evidence of the positive effects of interprofessional education (IPE) both on students' education and in the healthcare system [3, 4].

IPE occurs “when students from two or more professions learn with, from and about each other to improve collaborative practice and the quality of care” [5]. Thistletwaite [6] states that the prepositions “with, from and about” are central in interprofessional learning, and this means that all three prepositions must be applied in pedagogical practice when bringing different professional groups of students together in the same educational setting. To gain knowledge about other health professions, the students need to interact with each other across professional boundaries during their educational programmes.

Primary healthcare is usually the first level of healthcare contact for most individuals in the community, and an increasing number of health education programmes include primary healthcare in their curricula [7]. Although primary healthcare might be an optimal setting for IPE, it is often a challenge to organise IPE activities for students in primary healthcare due to the varying schedules and high demands on supervisors that have both clinical duties and supervising responsibilities [8]. This makes e-learning tools such as virtual patients (VPs) a practical and attractive complement to such activities, and VPs have been applied in medical education for more than a decade [9–11]. What constitutes a VP has been defined in a number of ways during the years, but one of the common definitions is the one by Ellaway et al. [12] “an interactive computer simulation of real-life clinical scenarios for the purpose of healthcare and medical training, education or assessment”. However, as pointed out by e.g. Kononowicz et al. [13] there might be other types of systems that are described as a “VP”, including the application of human actors and/or manikins. Kononowicz et al. conclude that “Interactive Patient Scenarios” is the most common use of the term, which is in concordance with Ellaway et al's definition above. A VP classified as an “Interactive Patient Scenarios” opens up for a rather large range of different functionalities and educational designs. VPs can facilitate learning in a safe and realistic environment and can provide opportunities for students to practice repeatedly without any risk of harm to the patient [12]. VPs are in most cases designed for only one specific health profession with the aim of teaching clinical reasoning skills and supporting professional development [10, 14, 15], and the use of VP activities in the fields of nursing and medicine have been extensively studied [16, 17]. We have found only one study that assessed a VP, that was aimed to be used by three different health professions [18], and we have not found any studies aimed at assessing the impact on students' learning of a VP created for IPE for students in a primary healthcare context.

The aim of the present study was to develop and assess a VP model designed for IPE in the context of primary healthcare. We also wanted to investigate students' perceptions of learning with a VP case in this model.

## Methods

### A virtual patient model for primary healthcare

We developed a VP model to support IPE and embedded within it a longitudinal VP case for this study. The model was designed from social constructivist and experiential learning theories [19, 20]. We aimed to create a VP model where the students construct their knowledge when interacting with students from other professions in a social context. The VP model was aimed to promote self-directed learning [21, 22]. The intention was to embed reflection-in-action in every learning cycle and to give the students an opportunity to reflect on their actions at the end of the session [23]. We assessed the VP model with interprofessional student groups and evaluated it through group interviews and a questionnaire with the participating students. Oral and written informed consent was obtained from all participants.

**Fig 1:**
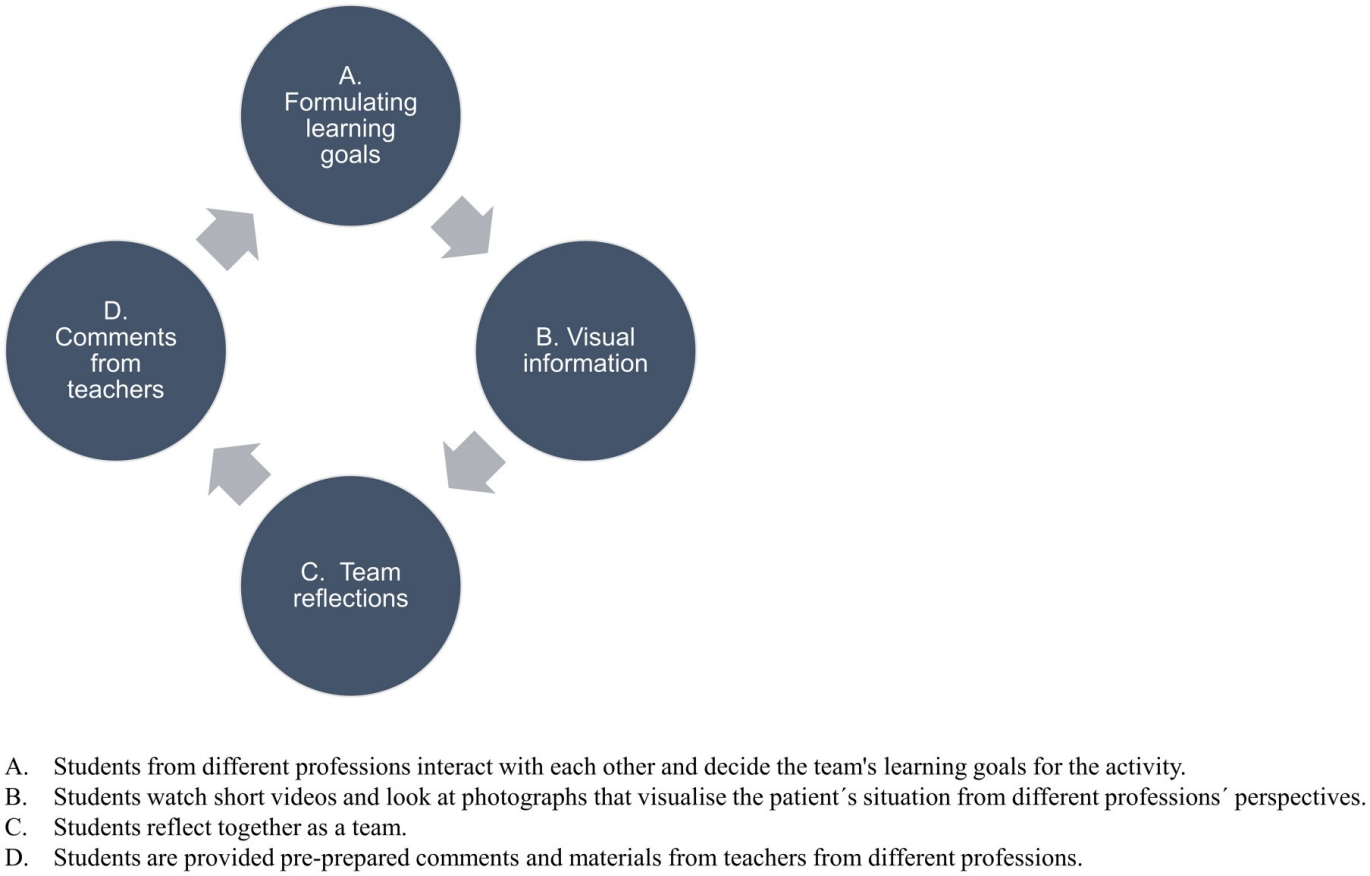
Interprofessional virtual patient learning cycle.

The interprofessional VP model (Fig 1) was based on a previously published VP model designed for medical students in Swedish primary healthcare [24]. The VP model was inspired by Kolbs' learning cycle [20], in that it contained all four of the stages: Concrete experience, Reflective observation, Abstract conceptualisation and Active experimentation. In the VP model, though, the stages were not distinct actions, but in some of them students did several activities. At the start of the VP students reflected on and summarised their learning goals for the learning activity. Then they were presented with the patient case, answered preparatory questions and watched a video clip (Concrete experience). In the next step, they reflected on the video clip and other provided written information (Reflective observation). In the third step, students reflected on the teachers' comments and compared it with their submitted answers in the previous step (Abstract conceptualisation). Lastly, they took along the summary of the first cycle to the next one where new situations, questions and videos were presented (Active experimentation).

The VP system was based on a previously developed e-learning platform called BSAsim. However, because the IPE VP case had the possibility to follow a patient over time and to navigate backwards or forwards in the time sequence, this platform needed several major adaptations. These included the opportunity for free text comments on all user inputs, providing video clips, and supporting a case that develops over time. However, the IPE VP system, still allowed a number of “classical” VP features including interactive illness history taking, physical exam, laboratory findings and imaging tests.

### Development of the interprofessional virtual patient case

The VP case embedded in the VP model was based on a modified authentic patient history in which a 73-year-old patient was receiving home care. The case was thoroughly reworked and changed so no individual patient, family member or healthcare worker could be identified from the VP case. We changed details such as name, age, family situation, additional diagnoses and medical test results. He had recently returned to his home from the hospital after surgery for a hip fracture. The patient also had multiple comorbidities including diabetes, congestive heart failure, and mild depression. The patient case was first scripted by a district nurse and a resident physician who both worked clinically in primary healthcare. We included other relevant professions' perspectives in the VP case by involving an occupational therapist, a physiotherapist, a counsellor, and a dietician. They all received the patient case via email and then were invited for discussions about how their professions could provide care for this patient. The compiled VP script and then the interactive case were revised by the whole research group consisting of family physicians, district nurses, and a psychologist. In the final version of the VP case, the students could follow the patient over three weeks of his at-home rehabilitation when the students were working with the VP case in a two hour session.

### Context and participants

District nurses and family physicians are the most common healthcare professions working at primary healthcare centres in Sweden, while occupational therapists and physiotherapists work at rehabilitation units. A district nurse is a specialist in community and primary healthcare who works with a high level of independency. When practicing in primary healthcare, students are placed at the unit where their supervisors work.

Ten sessions with a total of 39 students were conducted at two primary healthcare centres in Stockholm. The students came from four different study programmes, including nursing, physiotherapy, medicine, and occupational therapy. This study used a convenience sampling. Approximately 30–40 personal invitations were sent to students who were in their clinical placements. Information about the study was also disseminated through teachers and clinical supervisors. Additionally, ads were placed on campus and at the gym. The nursing, occupational therapy, and physiotherapy students who participated were in their fourth of sixth semesters, while medical students were in their third to ninth of eleven semesters (Table 1).

**Table 1:** The participating students in the study grouped according to profession, gender, age, experiences of IPE, and the semester they were studying.

	Student category				
	Total (N = 39)	Medical (N = 12)	Nursing (N = 16)	Physiotherapy (N = 4)	Occupational Therapy (N = 7)
Gender (M/F)	13/26	7/5	3/13	2/2	1/6
Median Age (years)	28	28	32*	32	26
No Experience of IPE**	10	4	5	-	1
Semester 3	1	1	-	-	-
Semester 4	15	-	9	1	5
Semester 5	12	2	6	2	2
Semester 6	3	1	1	1	-
Semester 7	5	5	-	-	-
Semester 8	1	1	-	-	-
Semester 9	2	2	-	-	-

*Information about age was missing for one student

**IPE = interprofessional education

### Data collection

The study was conducted from March 2016 to April 2017. The students worked with the VP case in face-to-face sessions that were limited to a maximum of two hours. Each student participated only once. Two out of the ten student groups had all four student professions, while two groups comprised only of nursing students. One group had three professions and the rest had only two. The students were asked to answer a questionnaire after they had completed the session. The questionnaire contained background questions and six questions about how the students perceived working with the program (S1 Table). There was also space for free-text comments and suggestions for improvement. After each session, the students were asked to participate in a group interview. Each interview lasted between 20 to 30 minutes using an interview guide with open-ended questions: Please tell me what it was like to learn together using the virtual patient? How did the virtual patient help you in learning about other professions? Please give some examples of what you learned about other professions. The interviews were conducted by the first author (CT), and were tape-recorded and transcribed verbatim.

### Qualitative analysis

The transcripts were analysed with qualitative content analysis following the guidelines of Graneheim and Lundman [25], and an inductive approach was used because there was no previous knowledge about how students perceived learning with this VP model. The data analysis started with a thorough reading of the transcribed interviews to get a sense of the whole material (CT, ETP, and HS), and the transcripts were coded independently by the same three authors. We coded the material manually. In accordance with the study's aims, the meaning units, i.e. constellations of words or statements that related to the same central meaning, were identified and derived from the data. The meaning units were then condensed and labelled with a code, and the codes were compared and those with similar content were grouped into sub-categories and then into main categories. In the next step, the same three authors analysed and discussed the results until consensus was reached. To illustrate these stages of the analysis, we have given examples of meaning units, condensed meaning units, codes and sub-categories under one of the main categories in a table (S2 Table).

### Ethical considerations

This study was approved by the Regional Ethical Review Board in Stockholm (2013/2267-31/4). All participants gave their informed consent prior to inclusion in the study, and their participation was on a voluntary basis and they could withdraw from the study at any time without explanation, and with no consequences for their education. Their anonymity and confidentiality were ensured. All the roles in the video clips were played by colleagues from different professions, including the patient and his wife. All individuals in the screen shots in this manuscript have given their written informed consent (as outlined in PLOS consent form) to have the photos from the films published.

No one from the research group was involved in teaching the students who participated in the study.

## Results

The VP model was assessed by 39 students in total from four different study programmes (Table 1). There were 26 female and 13 male students, and they ranged in age from 20 to 46 years old with a median age of 28 years.

### The VP model for interprofessional learning

**Fig 2:**
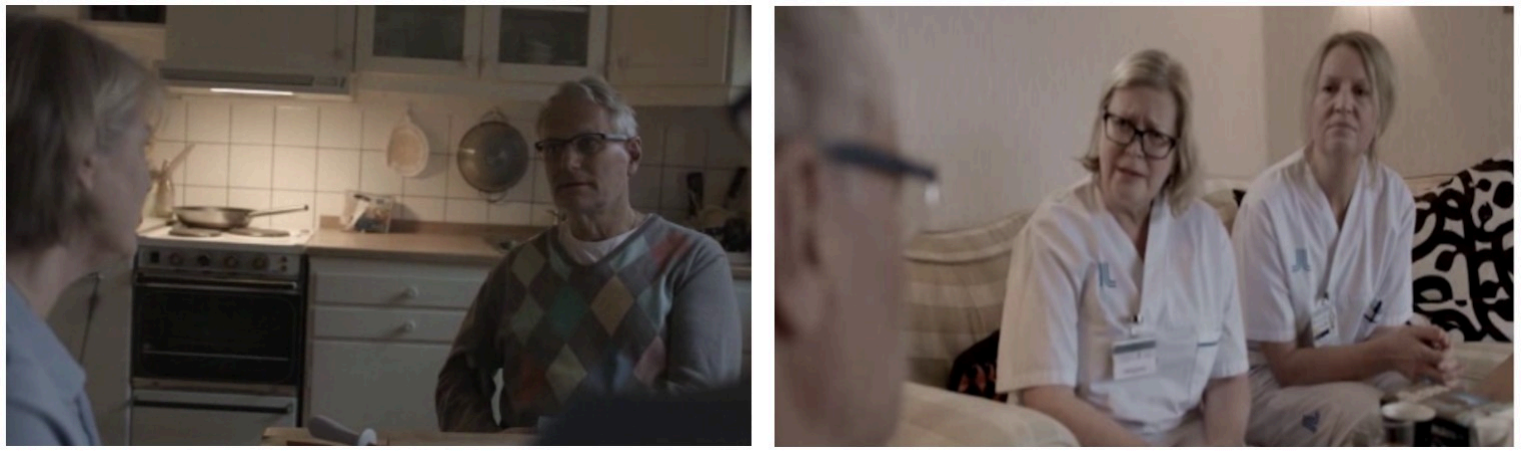
Screenshots from the video clips showing home visits by three different health professions—An occupational therapist on the left and a family physician and district nurse on the right.

All students were introduced to the learning objectives for IPE for all programmes at the medical university in the beginning of the VP model, before they formulated their own learning goals. The students were asked to reflect together on what they already knew and what knowledge they needed to expand on and after coming to consensus on learning goals, the group could start working with the VP case. The case included three short video clips that described the patient receiving home visits by four different health professions (Fig 2) and community-based home care. The film sequences also highlighted different aspects that were important in the VP case, such as medicine packets on the table and thresholds between rooms in the apartment that might be an obstacle to the patient. There were photographs showing an almost empty refrigerator and other signs of problems in the household. There were additional medical data about the patient to review, including an X-ray indicating congestive heart failure. The students could get more information by choosing among specific questions about the patient to which they got pre-prepared answers. The results of several assessment tools used by different professions and applicable for the patient case were available. The students could follow links that led to descriptions of the roles of all professions relevant to the patient case. Before and after each video clip the students were asked to discuss some questions in their group and then to write down their team's reflections. Each time the students had submitted in the system such a team reflection, they received feedback presented as pre-prepared comments from the multidisciplinary teaching team.

The students provided feedback about their perceptions from working with the VP model both in the interviews and in the questionnaire. The answers in the questionnaire showed that the majority of the students perceived working with the VP very positively (S1 Table). Though they had several suggestions for improvement. This feedback resulted in some changes in the VP. We restructured some texts into shorter paragraphs and reformulated some instructions to be clearer. We also improved the navigation in the system.

### Students’ perceptions of interprofessional learning with the VP model in primary healthcare

The qualitative content analysis of the group interview data sought to describe the students' perceptions of interprofessional learning when working with the VP and resulted in the following four main categories: 1. The VP model facilitated the learning process; 2. It was beneficial to have students from several professions in the group when working with the VP; 3. Working with the VP model helped the students to understand the roles and competences of their own and other professions; and 4. All professions are needed in clinical work in order to help the patient. The four main categories and all sub-categories are found in Table 2.

**Table 2:** Four main categories and 16 sub-categories that emerged from the text from the ten group interviews with the participating students. Number (%) of meaning units that constituted each main category.

Sub-categories	Main categories
The films make it easier to understand the patient's need for help Appreciate pre-prepared feedback from the teachers VP* flow clearly shows IPE** VP model requires basic knowledge	The VP model facilitated the learning process 128 (40%)
Learn more by working with VP together Require same professions as in the VP model Stimulate discussions and reflection Supplement each other's knowledge	It was beneficial to have students from several professions in the group when working with VP 36 (11%)
Realise one's limits Be able to hand over responsibility Provide new knowledge about other professions Combination of text and images made learning about other professions easier Virtual colleagues	Working with the VP helped the students to better understand the roles and competences of their own and other professions 120 (37%)
Reminded of significance of cooperating Can focus on one's own when working together Good basis for IPE	All professions are needed in clinical work in order to help the patient 38 (12%)

*VP = virtual patient

**IPE = interprofessional education

In order to illustrate how students perceived interprofessional learning with the VP model, anonymised quotes are included below.

#### 1. The VP model facilitated the learning process

All of the students reported that the VP model stimulated their discussions and reflections within the student group. Some students perceived that the VP was a reminder for them about situations when they should ask for help, and it exemplified what they could get help with from other professions. Having several options for learning, e.g. by watching, reading, and writing reflections, was perceived to consolidate their knowledge. They appreciated the video clips because they made it easier to understand the patient's needs for physical aids in his home. The structure of the information in the VP, with both text and photographs gave in-depth knowledge about the competencies of the professions involved. The students perceived that the comprehensive information about the patient provided a good overall picture of the patient's situation, but it also meant that the students needed to prioritise the different measures. Most students appreciated the prompt feedback presented as pre-prepared comments from the teachers because they filled knowledge gaps. Almost all of students perceived the patient case to be authentic, although some felt that the end was a little bit too optimistic because seldom are all problems resolved in real life. The students reported that it was clear and easy to understand how they were expected to work with the VP.

“It was very nice with the videos because then you see things a little clearer. You see the interaction more clearly, you see how they move, you see the home environment. It was actually kind of fun!”

(Nursing student, group 3)

“It was good to first write down one’s reflections, [and then] to see the teacher’s comments”

(Physiotherapy student, group 10)

#### 2. It was beneficial to have students from several professions in the group when working with the VP

The students said that they learned more when they could work with the VP together with other students from several different professions. The students who had only two or three professions in their group commented that the optimal situation would have been if all four professions shown in the VP case had also been present in their group of students. However, they expressed that having two professions in the group was better than only one. The students from only one profession in the same group reported that they perceived the VP less stimulating than the other students. When several professions were present, the discussions were more rewarding because they could ask questions directly to the students from other professions. The students felt that they confirmed each other's clinical reasoning and supplemented each other's knowledge when they worked together. To "think together" and discuss things made it easier to solve the VP case, and they felt that those discussions were more rewarding and more important than the answers they wrote to the questions posed as part of the VP model. The students enjoyed having the opportunity to work with professions that were less familiar to them.

“But it can be good to have all four professions involved in the case because then … you get even more to do”

(Occupational therapy student, group 4)

“I also thought that the discussion itself was what was the most rewarding; I almost think that what we wrote was a bit insignificant.”

(Medical student, group 10)

#### 3. Working with the VP helped the students to understand the roles and competences of their own and other professions

Several students reported that they got to better understand their own profession's boundaries. Some also felt a sense of relief in being able to entrust another profession with part of the responsibility for the patient, which was a new experience for them. They also found it inspiring when they discovered that they had different perspectives on the same matter. Most often it was the physiotherapists' and occupational therapists' perspectives that were new to nursing and medical students and vice versa, while physiotherapist and occupational therapist students and nursing and medical students had more familiarity about each other's respective roles. Several students reported that they gained new knowledge about the other professions and that the division of responsibilities became clearer. As one student expressed it: "Working with the VP was like having virtual colleagues".

“We see different things when we watch the video. I can see that he is out of breath and you see that he is tripping over the edge of the carpet.”

(Medical student, group 5)

“I thought these explanations of what the various professions did was good. Like, you could click on the care manager, what do they do, or you could click on the counsellor what do they do. It might make one curious to read a little about that profession.

(Occupational therapy student, group 7)

#### 4. All professions are needed in clinical work in order to help the patient

When working with the VP, students were reminded of the importance of collaboration with other professions in order to satisfy all of the patient's needs. They felt that they could focus more on their own profession's tasks when they had several different professions in the group, and they realised how much it helped when they collaborated. Several students also thought that the VP was a good foundation for learning to work as a team. The VP was also perceived as being a valuable complement to real life assignments and that it was especially useful for students who did not have the opportunity to take part in teamwork during their clinical practice. The students reported that working in teams with the VP increased their understanding of how to use each other's competencies when helping the patient. Students who thought they had some previous knowledge about teamwork also felt that they gained a better understanding of the importance of collaboration for improving healthcare and how collaboration could benefit the patient. Students also described how the complexity of the VP case made it necessary to discuss issues and to seek help from each other because none of them could satisfy all of the patient's needs on their own.

“That you have to work together to be able to meet all of a patient's needs.”

(Physiotherapy student, group 6)

“Although it was nothing new, there was an increased understanding of how important collaboration is.”

(Nursing student, group 2)

## Discussion

In this study we developed and assessed a VP model for interprofessional learning in primary healthcare. The results show that the VP model stimulated students to learn more about their own and other professions' competencies and helped them to better understand the importance of working in teams in clinical practice. The main finding was that the VP model was perceived by the students as a facilitator in their learning process in this interprofessional context.

Our intention in developing the VP model was to make the students actively reflect together before they answered the questions. The students perceived the VP to be a positive learning activity and found it to be a good foundation for practising teamwork. The VP model contained a lot of information, so the students had to prioritise their measures. We chose a patient with a hip fracture who needed medical, nursing, and rehabilitation help in his home after surgery, but this VP model is applicable for other patient cases as well. The first part of the VP described the family physician's and the district nurse's roles in homecare. This at first made it unclear for some of the students from other professions that the case was supposed to be solved by all of them as an interprofessional team. This was adjusted by adding this information to the text in the introduction before the start of the VP case.

The video clips in our VP model were used to demonstrate how different professions work together in homecare, and supportive information was presented on text pages and additional material via links in the system. This might explain why the students reported exclusively positive impressions about the video clips, in contrast to a study by Woodham et al. [26] where video clips were used to present all of the information. In that study the students felt that video was best suited to demonstrate procedures, and they suggested the use of a mixture of video and text for best results, which is how we designed our model. Watching a video clip might facilitate a better understanding of complex situations and procedures and illustrate the tools used by different professions. These findings are in accordance with a study by Peddle et al. [17]. Woodham et al. [26] found in their study that the students appreciated the video clips because they perceived that they made the scenario feel more real and the case more engaging, and we made similar observations in our study.

Peddle et al. [17] found “groupthink” to be a limitation to learning when only nursing students worked with a VP. Students in their study group influenced each other to express their thoughts more as a group than as individuals. In our study, the students came from different professions, which seemed to prevent groupthink, possibly because there was no competition between them and instead, they needed help from each other in order to solve the patient case. The students in our study reported that they enjoyed the interprofessional discussions. Working with the VP seemed to be a rewarding experience for both the students who needed to learn more about other professions and those who already had some knowledge about teamwork. Students in our study reported that they picked up new knowledge about other professions, which is in accordance with the study of Shoemaker et al. [18] where the students also reported having gained new knowledge about other professions when working with a VP. Most of the students in our study had some experience of collaboration with students from other professions, but they still had rarely collaborated with students from other professions during their clinical placements. In a previous study we found that students' tunnel-vision focus on their own profession often causes them to miss opportunities to collaborate with students from other professions [27]. In line with our findings, medical students in the study by Shoemaker et al. [18] also reported that sometimes they were caught up with focusing only on the medical aspects of the case, but working with the VP reminded them that it was important to collaborate with different professions.

The VP model made the students in our study realise that working with other professions also resulted in unexpected benefits. For example, they learned the importance of entrusting other professions to take care of specific issues in the case, and by learning to share the responsibility for the patient they also learned more about their own profession. It is possible to further expand the VP in the future by adding other healthcare professions. In our case we have chosen those professions that commonly work in primary healthcare in Sweden.

The categories from the interview analysis in the present study relate well to the four core competencies for interprofessional collaborative practice suggested by Interprofessional Education Collaborative [28]. In the category “*The virtual patient model facilitated the learning process*” the students described their perceptions on how the VP model facilitated their interprofessional communication and their learning about the importance of teamwork in patient-centred care.

A systematic review and meta-analysis by Kononowicz et al. [29] found evidence for virtual patients being underrepresented in other disciplines than in medicine and recommends further research regarding different design of virtual patients. The interprofessional VP model used in the present study was constructed with a framework of learning theories promoting self-directed experiential learning in interaction with other students. The VP model corresponds well to the demands of how to develop new virtual learning tools.

Our VP model was designed for blended learning, and the students interacted face-to-face when working with the case. It would be interesting to study if the students could benefit of this VP model when they work together in a distance learning context.

### Strengths and limitations

One strength of this study was that all four professions acting in the VP case were represented in several of the student groups working with the VP. In total we had 39 students from four different study programmes who evaluated the VP, and we consider that saturation was obtained. Another strength was that all professionals who contributed to the VP case were also actively practicing clinicians or teachers. This increased the authenticity of the patient case in the VP model and in the pre-prepared comments given to the students. The research group was also from different health professions, and the various professional backgrounds might have contributed to a variety of perspectives on the studied topic.

One limitation was that some of the participating students had not yet had their own experience in the provision of homecare, which might have limited their possibility to contribute to the group discussions when working with the VP. Another limitation could be that two of the groups had only two participating students from the same profession. Additional limitations include that the study was conducted at only one medical university with one cohort of participants who only met once. On the other hand, this is one of the common scenarios in which we expect the VP to be used in education in the future.

## Conclusions

The findings in this study showed that the VP model facilitated interactions and discussions between students, and the students valued the comprehensive information about different professions' roles via the video clips and written texts. Thus, the VP model presented here might be a suitable tool for preparing students for future teamwork in clinical practice.

## Supporting information

S1 Table Students' answers to six questions about their perceptions of using the program with the VP. Answers were on a 6-graded Likert scale (1 = Strongly disagree; 6 = Strongly agree).
(DOCX)

S2 Table Illustration of the process of analysis with examples of meaning units, condensed meaning units, codes and sub-categories building one of the main categories. (DOCX)
