# Supplementary material for: A virtual patient model for students’ interprofessional learning in primary healthcare
Source: PLoS One. 2020 Sep 23;15(9):e0238797. doi: 10.1371/journal.pone.0238797 (PMC7511020; doi:10.1371/journal.pone.0238797)
Supplement: S1 Table — Answers were on a 6-graded Likert scale (1 = Strongly disagree; 6 = Strongly agree). (DOCX) [file pone.0238797.s001.docx]

**S1 Table.** Students' answers to six questions about their perceptions of using the program with the VP. Answers were on a 6-graded Likert scale (1= Strongly disagree; 6= Strongly agree).

|  | **Student category** | | | | |
| --- | --- | --- | --- | --- | --- |
|  | **Total**  (N=39) | **Medical**  (N=12) | **Nursing**  (N=16) | **Physio-therapy**  (N=4) | **Occupational**  **Therapy**  (N=7) |
| Question | **Median**  **(min, max)** | **Median**  **(min, max)** | **Median**  **(min, max)** | **Median**  **(min, max)** | **Median**  **(min, max)** |
| Q1. The program was easy to use. | 4  (1, 6) | 4,5  (2, 6) | 4,5  (1, 6) | 4  (4, 5) | 3  (3, 5) |
| Q2. I would have appreciated more help to learn how to use the program. | 2  (1, 6) | 1  (1, 5) | 2  (1, 6) | 2,5  (2, 4) | 2  (1, 5) |
| Q3. It was fun to use the program. | 5  (2, 6) | 5  (2, 6) | 5  (3, 6) | 3,5  (3, 5) | 3  (3, 5) |
| Q4. It was easy to understand how to navigate in the program. | 5  (2, 6) | 5,5  (2, 6) | 6  (2, 6) | 4,5  (3, 5) | 4  (2, 6) |
| Q5. It was easy to understand what you were expected to do in the program. | 4  (1, 6) | 4,5  (1, 6) | 5  (2, 6) | 3,5  (2, 4) | 3  (2, 6) |
| Q6. The program had a pleasant design. | 5  (2, 6) | 4  (2, 6) | 5  (2, 6) | 4,5  (4, 5) | 4  (3, 6) |
