# Supplementary material for: A virtual patient model for students’ interprofessional learning in primary healthcare
Source: PLoS One. 2020 Sep 23;15(9):e0238797. doi: 10.1371/journal.pone.0238797 (PMC7511020; doi:10.1371/journal.pone.0238797)
Supplement: S2 Table — (DOCX) [file pone.0238797.s002.docx]

**S2 Table.** Illustration of the process of analysis with examples of meaning units, condensed meaning units, codes and sub-categories building one of the main categories.

| Meaning unit | Condensed meaning unit | Code | Sub-categories | Main category |
| --- | --- | --- | --- | --- |
| ... and then you need to formulate it for your self ... what am I supposed to do, what is my role and what belongs to the role of a physician or a nurse in the actual situation? (group 5, PT, T5)* | You need to formulate your role for yourself | A need to formulate one's role | Realise one´s limits | Working with the VP helped the students to better understand the roles and competences of their own and other professions |
| **...** you are always looking through your profession's glasses, and you may have missed something that someone else [another profession] catches instead (group 8, N, T4) | You may have missed something that another profession catches | Another profession notices things that I don't |  |  |
| I will be ... as a nurse, working with all those professions, and it will be easier for me if I know who has the responsibility [for a task] and who doesn't. (group 6, N, T5) | This makes it easier for me to know who has the responsibility | To know who has the responsibility | Be able to hand over responsibility |  |
| ... as a medical student I think that I have a general idea of the whole situation, but I can feel relieved, when it concerns physical rehabilitation, that I can hand over to a physiotherapist. (group 5, M, T7) | It's a relief to be able to hand over physical rehabilitation to a physiotherapist | Some tasks you have to hand over to other professions |  |  |
| You discover all those things you don't think about in your profession ... there are plenty of things that a physician or a nurse do, but I as an occupational therapist don't, and I haven't even reflected about it before (group 4, OT, T4) | Discovers new things about other professions and reflects about other professions skills | Discovers new things about other professions | Provide new knowledge about other professions |  |
| I have now understood that eh ... a physiotherapist, it is more physical ... how ... eh ... exercise. Physical exercise, while occupational therapist is more ADL (Activities of Daily Living). (group 8, N, T4) | I have now understood that physiotherapy is more focused on physical exercise, whilst occupational therapy is more focused on ADL. | Understanding what physiotherapists and occupational therapists do |  |  |
| Because you could see [on the videos] when the different professions entered [in the patient's home] and what they did there, and that is the most important (group 8, M,T9) | You could see what different professions did, and that is the most important | To see what different professions do | Combination of text and images made learning about other professions easier |  |
| I thought these explanations of what the various professions did was good. Like, you could click on the care manager, what do they do, or you could click on the counsellor what do they do. It might make one curious to read a little about that profession. (group 7, OT, T5) | It was helpful to read about what different professions do | To read about different professions |  |  |
| It [the VP] stimulates reflection very much . Because, on the clinical placements you always have a supervisor who guides you. Here you really have only your own reflections when you work with students from other professions. (group 9, N, T5) | Here you have to rely on your own reflections when working with students from other professions. | One's own reflections together with other professions | Virtual colleagues |  |
| It [the VP] helps to understand those [professions] who are not here, without them being present [in the student group]. We can see what they can do ... It is like having virtual colleagues.  (group 2, M, T3) | It is like having virtual colleagues | Virtual colleagues |  |  |

M=Medical; N=Nursing; PT=Physiotherapist; OT=Occupational therapist
